# The mechanical, optical, and thermal properties of graphene influencing its pre-clinical use in treating neurological diseases

**DOI:** 10.3389/fnins.2023.1162493

**Published:** 2023-06-09

**Authors:** Ting Ye, Yi Yang, Jin Bai, Feng-Ying Wu, Lu Zhang, Long-Yue Meng, Yan Lan

**Affiliations:** ^1^Department of Physiology and Pathophysiology, College of Medicine, Yanbian University, Yanji, Jilin, China; ^2^Interdisciplinary Program of Biological Functional Molecules, College of Intergration Science, Yanbian University, Yanji, Jilin, China; ^3^State Key Laboratory of Medical Neurobiology, Institutes of Brain Science, Fudan University, Shanghai, China; ^4^Department of Environmental Science, Department of Chemistry, Yanbian University, Yanji, Jilin, China

**Keywords:** neuroscience, nanomaterials, graphene, neurons, treatment

## Abstract

Rapid progress in nanotechnology has advanced fundamental neuroscience and innovative treatment using combined diagnostic and therapeutic applications. The atomic scale tunability of nanomaterials, which can interact with biological systems, has attracted interest in emerging multidisciplinary fields. Graphene, a two-dimensional nanocarbon, has gained increasing attention in neuroscience due to its unique honeycomb structure and functional properties. Hydrophobic planar sheets of graphene can be effectively loaded with aromatic molecules to produce a defect-free and stable dispersion. The optical and thermal properties of graphene make it suitable for biosensing and bioimaging applications. In addition, graphene and its derivatives functionalized with tailored bioactive molecules can cross the blood–brain barrier for drug delivery, substantially improving their biological property. Therefore, graphene-based materials have promising potential for possible application in neuroscience. Herein, we aimed to summarize the important properties of graphene materials required for their application in neuroscience, the interaction between graphene-based materials and various cells in the central and peripheral nervous systems, and their potential clinical applications in recording electrodes, drug delivery, treatment, and as nerve scaffolds for neurological diseases. Finally, we offer insights into the prospects and limitations to aid graphene development in neuroscience research and nanotherapeutics that can be used clinically.

## 1. Introduction

Nanotechnology has nanometric resolution referring to the length of a billionth of a meter (Linda and Wade, 2017). This engineering technology allows device manipulation to a microscopic structure that suits the basic orders of biomolecule systems. With their controllable nanoscale properties, scientists have exploited various synthetic materials and devices for treating and monitoring pathological conditions in biomedicine.

A size change in a nanostructure induces variations in its functional properties (Berube, 2006), showing great potential for evaluating and solving several neurological problems, from neurons to neurological diseases. Nanomaterials can be used to construct biomimetic neural networks *in vitro* because of their physical and chemical properties (Malarkey and Parpura, 2010). Moreover, nanomaterials can be used for targeted drug delivery *in vivo* with minimal side effects through the fabrication method to improve the efficacy of cancer chemotherapy and enhance the efficiency of magnetic resonance imaging-guided gene delivery as radiation sensitizers and contrast enhancers (Nezakati et al., 2014). Driven by the lack of precise diagnosis and therapeutic strategies in the clinic (Shi et al., 2013), the development of neural nanotechnology and nanomaterials used for studying the mechanisms, diagnosis, and treatment of nervous system disorders has to breakthroughs in using carbon nanomaterials.

Two-dimensional (2D) materials have been extensively explored for biomedical applications (Convertino et al., 2020b). With super structural rigidity and a good surface-to-volume ratio, 2D materials provide maximal interaction between the environment and surfaces, allowing for high sensitivity and rapid performance with small sample volumes (Palumbo et al., 2018). Andre Geim and Konstantin Novoselov studied graphene and successfully separated it from graphite in 2004 (Novoselov et al., 2004). Graphene is the most widely studied nanomaterial for cell-based device platforms with peculiar properties, including its role as a polymeric conduit for nerve regeneration, carrier for targeted drug delivery, and for photo-thermal cancer therapy. Recently, highlighting the role of graphene in biomedical fields has inspired great interest in its analogs, such as transition metal dichalcogenide (Convertino et al., 2020b). However, compared with other nanomaterials, including carbon nanotubes and transition metal dichalcogenide, graphene has higher mechanical strength, chemical stability, and specific electrochemical properties for electrical signal detection and transmission. Furthermore, it can be used as a conductive stent or biosensor in the medical field. These characteristics make graphene and graphene-derived materials excellent candidates for detecting signals in the nervous system.

Previous studies have shown that graphene can aid in the adhesion and differentiation of neurons and can act as a neuroprotective agent. Graphene is a potential carrier to control drug release at specific disease sites, making it a valuable treatment strategy for neuronal diseases. Moreover, graphene and its derivatives can be used as scaffolds to enhance nerve repairing, establishing it as a next-generation candidate for advancing medical bioengineering. However, the safety of nanomaterials is essential to protect human health and ensure environmental safety. Thus, further studies are warranted to fill the knowledge gap on the limitations of carbon nanomaterials, including their toxicity and biocompatibility (Nezakati et al., 2014).

In this review, we focus on novel graphene applications in the nervous system. Graphene-based materials may be used clinically in the future; however, this article is limited to pre-clinical applications. We aimed to describe the properties of graphene-based materials applicable in neuroscience research, including using them as electrodes for neural recording and imaging, neural cell substrates, photothermal effects, and drug and gene deliveries. Furthermore, we aimed to outline the application of graphene-based materials in neurological diseases and neural tissue engineering. Finally, we aimed to discuss graphene’s cytotoxicity and provide an overview of its prospects.

## 2. Roles of graphene in neuroscience

**Figure 1:**
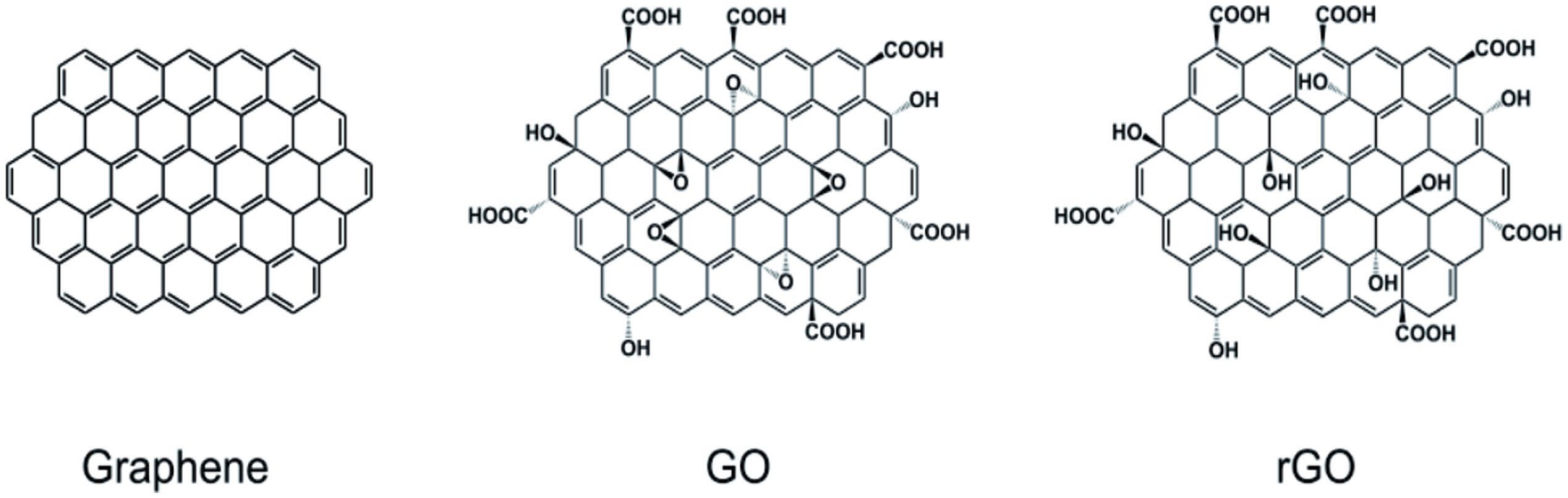
Structure diagram of graphene and other forms. (left) graphene; (middle) graphene oxide; (right) reduced graphene oxide. Kitko et al. (2018), Bramini et al. (2016), Ding et al. (2020), López-Dolado et al. (2016), and Serrano et al. (2018) with permission from Elsevier Ltd., copyright 2016.

Graphene is a sheet comprising a single layer of carbon atoms formed by stripping highly oriented pyrolytic graphite, like carbon nanofibers, another type of nanomaterial. In 2D, sp2 hybridized carbon atoms give graphene a hexagonal honeycomb lattice pattern with a thickness of approximately 0.335 nm [Figure 1 (left)]. Graphene normally can be synthesized by two main approaches, namely physical and chemical approaches. The chemical vapor deposition (CVD) technique has been commonly applied to produce large-scale, high-quality graphene film with no oxygen content, as well as defect-free hexagonal lattice (Reina et al., 2009). Hong et al. employed the CVD approach to generate large-scale and few-layer graphene films on a 300 nm-thick layer and then transferred them to a SiO2/Si substrate. Bea et al. developed monolayer graphene films of 30 inches by the CVD approach on a large copper substrate. The films showed low resistance of 125 Ω·m^−1^, superior to graphene grown on indium tin oxides. Unfortunately, this technique requires a very high temperature and vacuum environment, as well as exorbitant prices for only small-size devices. Many graphene-based nanomaterials are built up mostly by chemical reactions for covalent bonding, such as graphene oxide [GO; Figure 1 (middle)] and reduced graphene oxide [rGO; Figure 1 (right)]. The most popular approach to prepare graphene for bio-neurological studies is the chemical reduction of graphite GO to rGO, which involves severe oxidation of the material, followed by sonicated irritation to exfoliate the GO and reduction by chemical or thermal processes. Hummer’s method is widely used to produce GO by reacting graphite flakes with oxidizing agents/acids (Hummers and Offeman, 1958). In terms of health issues, especially in the biomedical field, green synthesis has gained considerable interest by using green reductants, such as L-ascorbic acid, D-glucose, and tea polyphenol (Xu et al., 2015).

At room temperature, the interactions between carbon atoms in graphene are strong. Electrons in the carbon atoms are not easily affected or scattered by the movement of surrounding carbon atoms; its conductivity also contributes to electron transfer (Bramini et al., 2016). Additionally, its large surface area and potential for binding different biomolecules onto its surface make graphene a suitable nanomaterial for holding small-molecule drugs, genes, proteins, deoxyribonucleic acid (DNA), and small interfering ribonucleic acid (RNA) (Mousavi et al., 2019; Ma et al., 2014). In neuroscience, the electrical conductivity, biocompatibility, and mechanical, thermal, and optical properties of graphene (Table 1) are its most important characteristics. Graphene can enhance the properties of nanomaterials for various biomedical applications, including electrodes for neural recording, stimulation, and imaging (Sun et al., 2008; Wei and Wang, 2021), neural cell and tissue scaffolding (Lee et al., 2011), photothermal therapy (PTT) (Robinson et al., 2011; Yang et al., 2012a; Peng et al., 2023), and drug delivery (Mauro et al., 2020; Dash et al., 2021). Recently, researchers have discovered new applications of graphene in nerve tissue engineering (Magaz et al., 2021) and neurological diseases, such as brain tumors, Alzheimer’s disease (AD), and Parkinson’s disease (PD) (John et al., 2015; Cho et al., 2022). These properties and applications suggest that graphene has a promising role as a biomaterial in neuroscience.

**Table 1:** The applications of graphene properties in neural areas.

Property of graphene	Form of graphene	Applications	References
Mechanical properties and electrical conductivity	Polyaniline-graphite electrode	Promotes damaged sites regenerate.	Zheng et al. (2019)
	Poly-3,4-ethylene dioxythiophene/graphene oxide composite film	It can be used an implantable device to modify the electrode site.	Ding et al. (2020) and Kitko et al. (2018)
Optical properties	Combine ventral window with graphene sensor	The activity of the enteric nervous system is monitored and recorded by light stimulation.	Rakhilin et al. (2016)
Thermal properties	Graphite lattice	Uses in drug delivery for neurological diseases.	Monaco et al. (2014) and de Melo-Diogo et al. (2018)
	nGO-PEG	The tumors in the body are drastically removed by near-infrared laser irradiation	Yang et al. (2012a) and Chen et al. (2013)
	Graphene	Photothermotherapy isolates the amyloid β-protein fibers in Alzheimer’s disease.	Li et al. (2013) and Bayer (2017)
Biocompatibility	Hydrogels containing graphene	Promotes neuron regeneration and support its differentiation.	D’Abaco et al. (2018), Javadi et al. (2018), and Liao et al. (2018)
	Graphene	Maintains the viability of neurons.	Fischer et al. (2018)
Toxic properties	Human umbilical cord Wharton’ s jelly-derived mesenchymal stem/stromal cells grown on reduced GO	Cells to show smaller and elliptical shape and differentiate into nerve cells.	Jagiełło et al. (2019) and Dybowska-Sarapuk et al. (2020)
	rGO	The survival rate of A549 cells are reduced.	Guo et al. (2014)
	GO	It leads to nervous system damage, increased risk of behavioral defects.	Guo et al. (2014), Chen et al. (2013), Li et al. (2017), Pattammattel et al. (2017), Ding et al. (2020), and Xiao et al. (2016).

nGO-PEG, polyethylene glycol functionalized nano-graphene oxide; rGO, reduced graphene oxide; GO, graphene oxide.

## 3. Mechanical properties and electrical conductivity of graphene in neural electrodes

Neural interfaces are typically implanted with electrodes that can record or stimulate target tissues or cells in the brain, thereby establishing connections with the nervous system (Keefer et al., 2008). However, current electrode materials and technology have limitations. The formerly used metal electrode has poor stability, high hardness, and greater electrical noise, and its insertion may lead to mechanical damage of neurons and surrounding soft tissues, obstructing electrophysiological recording (Szarowski et al., 2003). The sp2 bond structure provides graphene with high resistance to elongation, with Young’s modulus of 1,100 GPa and fracture strength of 125 GPa (Lee et al., 2008). Bending the graphene sheet does not result in considerable distortion of these bonds. Thus, graphene-based electrodes are perfect for touching soft samples like nervous tissues (Palermo et al., 2016). Young’s modulus ranges from 100 Pa to 10 kPa, which results in great tensile stiffness and strength while remaining highly flexible. Moreover, the unique structure of conjugated sp2 bond in the graphene plane excites electrons from the valence to conduction bands with near-zero energy, like the zero band gap nature of metal, giving graphene an outstanding electrical conductivity (Jiang et al., 2007; Li et al., 2009). With great electrical conductivity and high mechanical flexibility, graphene can be a suitable biomaterial as a neuronal interface for stimulating and recording brain signals.

Graphene can be mechanically deformed beyond the linear regime (Lee et al., 2008); this is particularly important for increasing mechanical compliance in *in vivo* microscopy techniques performed on freely behaving animals. However, one of the major problems of using graphene in *in vivo* studies is that it is ultra-flexible and difficult to implant surgically. Kuzum et al. (2014) fabricated graphene electrodes on flexible polyimide substrates, increasing its stiffness for implantation the brain without causing discomfort. Applying oxygen plasma etching to pattern graphene could also provide stiffness to the electrode, allowing it to penetrate brain tissues. An alternative method is using a stiff carrier microneedle that attaches the flexible electrode, aiding it to reach the desired depth (Kim T. I. et al., 2013). The microneedle is disengaged from the flexible substrate and withdrawn from the tissue once it reaches the required depth. This procedure only causes minimal invasive operation; however, the back-and-forth motion may increase the risk of inflammation during insertion. Researchers have recently exploited temporary stiffening of graphene electrodes with silk fibroin, which provides a rigid structure that reaches the desired depth (Weltman et al., 2016). The silk fibroin coating dissolves quickly after insertion, enabling the graphene electrodes to return to their original flexibility.

Unfortunately, scaling down the microelectrode dimension while sustaining a high signal-to-signal ratio (SNR) for recording cellular activity is challenging. Studies have demonstrated effective *in vivo* neural recording by graphene electrodes compared to gold electrodes (Kuzum et al., 2014; Park et al., 2014). In these studies, the relatively low capacitance of a few graphene layers led to a high impedance with large noise, which is unfavorable for maintaining high SNR in microelectrode recording. Common methods for enhancing graphene conductivity use chemical doping approaches, such as micropatterning, to increase the number of active sites. Qu et al. (2010) demonstrated that using graphene doping with nitrogen-containing compounds could increase the electroactivity of graphene-based electrodes. Moreover, researchers have fabricated graphene with conducting polymers, such as polystyrene sulfonate, polypyrrole, and poly-3,4-ethylene dioxythiophene; the synergistic effect increases charge transfer on the graphene surface. With great mechano-electrical stability, this electrode accurately recorded electrophysiological signals of brain activity with increased SNR (Zhao et al., 2021; He et al., 2022). Recently, Bonaccini Calia et al. (2022) designed a linear array of graphene neural depth micro-transistors to increase electrical conductivity and demonstrated that this microelectrode could concurrently and stably record direct current-shifts and high-frequency neuron activity in awake rodents, which is consistent with the results of Masvidal-Codina et al. (2021). By decreasing the impedance between graphene and electrolyte, Chen et al. (2013) fabricated a graphene surface with a mild steam plasma, thus increasing the SNR during neural recording.

The mechanical properties, electrical conductivity, and large surface area of graphene make it perfect material for developing ideal neural interfaces and more reliable electrodes, while highlighting the unique advantages of composites, demonstrating greater application potential in neural interfaces. However, more studies are required to investigate the long-term effects of graphene-based nanoelectrodes during *in vivo* recording validation.

## 4. The optical property of graphene in neural imaging and photothermal therapy

Traditional metal electrodes used for recording electrophysiological activities obstruct the vision field, produce optical shadows, and are more likely to introduce light-induced artifacts during the recording. Researchers have discovered that transparent graphene could solve this problem due to the unique band structure in the transmission spectrum of graphene that prevents the mentioned limitations. Theoretically, graphene allows the hot ballistic charge carriers produced by light (Gabor et al., 2011) to transfer their energy in a highly efficient carrier-to-carrier scattering process, with a mean path that can reach approximately 1 μm (Novoselov et al., 2004).

Graphene is highly transparent and absorbs 2.3% of incident visible light radiation (Kang et al., 2016), which allows for optimal optical access and avoids photoelectrical artifacts. These advantages are excellent in optical imaging applications, offering substantial improvements over conventional metal electrodes for the three-dimensional (3D) imaging application. Graphene enables spatial–temporal resolution for simultaneous imaging while recording physiological activity from the same target, especially for studying the wiring of neural circuits. Kuzum et al. (2014) recorded calcium fluorescence images via graphene electrodes in the dentate gyrus region without inducing artifacts from laser light. They observed overlapping neural cell bodies around the electrode while recording synaptic potential *in vivo*. Driscoll et al. (2021) used transparent graphene microelectrode arrays and successfully recorded simultaneous high-bandwidth electrophysiology and calcium imaging signals of brain network activity induced by epileptic seizures *in vivo*. Moreover, these cellular-scale methods enable the recording of electrophysiology and calcium fluorescence in CA1 pyramidal neurons in hippocampus seizures (Mulcahey et al., 2022). Moreover, inspired by the accomplishments of brain studies, integrating a transparent graphene sensor onto a designed abdominal window detected changes in the activity of the enteric nervous system through simultaneous optical and electrical recording in a live mouse (Rakhilin et al., 2016). From monitoring the activity of isolated neurons to forming intermuscular nerve plexus, this approach can help understand how the nervous system processes information. Thus, this technology can decipher and intervene in sequential spatiotemporal patterns of how the temporal progression of electrophysiology changes are associated with the spatial evolution of recruited core during brain disease onset and evolution.

Graphene exhibits a resonant response to any frequency photons in the ultra-broadband spectral spectrum from ultraviolet to infrared (IR) region (Wang et al., 2008). Recently, Savchenko et al. (2018) introduced a graphene-based optical actuator that uses light exposure to optically stimulate cells in a non-invasive manner, causing membrane depolarization. However, as a biomaterial, the optical absorption is low at 2.3%. Researchers have discovered that changing the physical architecture of graphene by increasing its flake or density can increase its optical absorption. For example, Rastogi et al. (2020) composed 3D fuzzy graphene with an out-of-plane shape, demonstrating great photothermal effects due to its high optical absorption in the near-IR (NIR) regime and non-invasively stimulating and mediating electrophysiological activity of neural spheroids. One practical concern about these optical stimulation methods is the possibility of off-target or adverse consequences due to the temperature increase in the surrounding neurons. Therefore, minimizing temperature fluctuations is preferable if the neurons are optically excitable. Anchoring graphene-based materials with appropriate biological ligands can specifically target different neural cells.

PTT has received widespread attention because it can thermally ablate cancer cells in a minimally invasive manner under an NIR laser. The broad optical absorption spectra are important in photothermal medical applications. Nanomaterials with strong absorption in the NIR regime can act as effective PTT absorbers that achieve therapeutic temperatures with less total light energy, reducing heat escaping from the target to healthy surrounding tissue and avoiding tissue damage. GO and rGO have been widely exploited as effective absorbers for NIR-based PTT in cancer therapy (Yang et al., 2012b; Akhavan and Ghaderi, 2013a). Clinical treatment requires specific and high-quality photothermal effects. Conjugating nanomaterials surface with modifying molecules, such as polymers and antibodies, can solve this problem. Yang et al. (2010) discovered that GO-polyethylene glycol (PEG) composite nanomaterials combined with the NIR fluorescent dye Cy7 could be successfully localized in target tumor *in vivo* and achieved ultra-efficient tumor ablation after laser treatment. Similarly, Akhavan et al. (2012) attached rGO-PEG with arginine-glycine-aspartic acid-based peptide and Cy3 to successfully target human glioblastoma cell line U87MG. This material exhibited high NIR absorption and resulted in 97% cell destruction *in vitro*. However, the rGO-PEG showed concentration-dependent cyto- and geno-toxicity. No toxic signal was found in the histological examination of GO-PEG during PTT by Liu operation. Another transition metal disulfide that is attracting more interest in the biomedical field is Tungsten disulfide because of its photothermal properties, high lubricity, and catalytic activity. Its high NIR absorption capacity has been applied to photothermal therapy of tumors (Appel et al., 2016). The development of graphene optical properties has promoted the *in vitro* and *in vivo* research of carbon nanomaterials (including graphene, carbon nanotubes, and diamond).

Photoacoustic and fluorescence imaging can also be combined with PTT for diagnosis. Photoacoustic imaging has few specific signals and cannot image deep tissues. Fluorescence imaging does not use ionizing radiation and has low tissue penetration. Thus, further research is needed to fully understand how to apply carbon nanomaterials better and fully in neurology, as their functions involve the interaction between their various properties. Therefore, improving the functionalization of nanomaterials, which can create better and less risky options for patients during clinical treatment of diseases, remains challenging.

## 5. Thermal property of graphene in photothermal therapy

Graphene has high thermal conductivity (κ) and great thermal management potential, indicating its development in nanoscale engineering for heat transport and management. Ma et al. (2017) found that the thermal conductivity of graphene significantly reduced with decreasing grain size by a thermal boundary of ~3.8 × 10^9^ W m^−2^ K^−1^. Kumar et al. (2015) reported that large-area GO produced a very high thermal conductivity of 1,390 Wm^−1^ K^−1^.

Researchers have suggested various effective approaches for modulating the thermal conductivity of graphene. Depositing nanoparticles on the surface of graphene can mediate its thermal management (Wang J. et al., 2013; Sadrolhosseini et al., 2019; Sun et al., 2022). Chemical functionalization, such as through small coverage of fluorine (Chien et al., 2011; Pei et al., 2011), hydroxyapatite (Nie et al., 2022), polymer (Wang et al., 2020, Kang et al., 2022, Lei et al., 2022, Tan et al., 2022), hydrogen (Zhang et al., 2010, Jarrahian and Heidaryan, 2012, Zhang and Liu, 2019), and phenol (Bernal et al., 2018), has been theoretically used to significantly enhance graphene’s thermal conductivity.

As thermal stability improves, graphene use has risen in the fields of clinical biomedical application and tailored nanomedicine for neurologic diseases. For example, graphite lattices in the graphene family of nanomaterials fuse to form disparate forms of molecules, with a potential role in drug and gene delivery for treating neurological diseases. Using two complementary strategies for conferring static or spatial stability can improve the thermal stability of graphene and allow cells to occupy stable graphene sheets (Hong et al., 2012). In 2008, Liu et al. (2008) first synthesized GO-PEG composite to deliver the anticancer drug SN38 into the cancer cell interior. Zhang et al. (2010) further conjugated GO with folic acid and loaded it with multiple anticancer drugs to specifically target human MCF-7 cells. In addition to the delivery of therapeutic molecules, GO-conjugated polyethyleneimine (PEI) loaded with small interfering RNA or plasmid DNA could be used to reduce its target gene expression (Feng et al., 2011; Kim et al., 2011; Zhang et al., 2011). Additionally, Barrera et al. (2020) functionalized rGO with iron oxide nanoparticle deposition, which increased thermal temperature. GO functionalized with ferrimagnetic vortex-domain iron oxide also showed high thermal conversion efficiency *in vivo* (Liu et al., 2020). The composite rGO-Fe_3_O_4_ increased the temperature by approximately 50°C in 5 min, which is considered the cell-killing temperature, meaning it can accomplish advanced PPT. Cao et al. (2017) reported a system with GODs-PEG and porphyrin derivate (P). A GQD-PEG-P solution of 100 μg/mL increased the temperature to 53.6°C while maintaining the temperature of control water at 33.2°C. GOD-PEG-P combined with PTT demonstrated excellent efficiency in killing cancer cells *in vivo* and *in vitro*. AD is characterized by neuronal loss in the cerebral cortex and subcortical areas and amyloid β-protein (Aβ) accumulation. Recently, Wang et al. (2021) demonstrated that GO loaded with dauricine could inhibit the aggregation and misfolding of Aβ. Moreover, graphene-based PTT can effectively dissociate the Aβ fibrils upon NIR laser irradiation (Li et al., 2012).

These findings provided new ideas for combining PTT and graphene-based drug/gene delivery therapy and demonstrated a significant improvement over traditional surgical treatment method. Graphene-based materials showed high potential for the novel and efficient treatment of neurological disorders. However, the interaction between graphene and cells may interfere with the different cell viability reduction rates in thermal therapy (Barrera et al., 2020). Future research should focus on the molecular and cellular effects of graphene composites in thermal and delivery therapy, given its promising results.

## 6. Biocompatibility and toxicity of graphene in neural interaction

The first aspect to consider when developing graphene-based biomedical applications is their biocompatibility to avoid any adverse effects on living tissues (Li et al., 2011). Graphene is highly biocompatible, making it an ideal choice for neural interface materials in biological tissues (Sahni et al., 2013; Fanizza et al., 2022). Furthermore, graphene can improve the efficiency of implants or scaffold materials to enhance cell differentiation and proliferation owing to its unique biocompatibility (Lee et al., 2011; Ulloa Severino et al., 2016; D’Abaco et al., 2018). However, the biocompatibility of graphene depends on its functionalization, which can also improve the biocompatibility of graphene-based materials. For example, chitosan is another biocompatible material, and its composite, chitosan-GO, showed better biocompatibility than GO scaffolds alone, with enhanced cell infiltration and reduced inflammatory reactions (Vlasceanu et al., 2020). Additionally, the hydrogen bond interaction between chitosan-GO composites can enhance the regeneration and activity of nerve cells (López Tenorio et al., 2019). Hydrogel-containing graphene are synthetic materials similar to living tissues and can promote neuronal regeneration and differentiation, which is valuable in developing synthetic materials for engineering neuronal tissues (Javadi et al., 2018).

**Figure 2:**
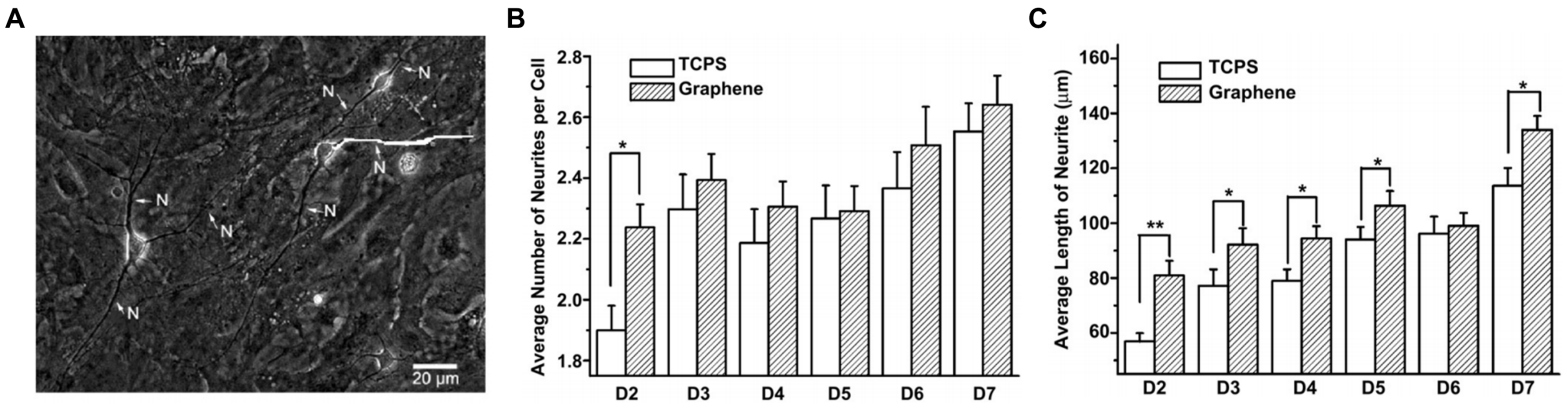
**(A)** Phase-contrast microscopic image of neurons. **(B)** The average number of neurites per cell on graphene and TCP. **(C)** The average length of neurite on graphene and TCP. Data are expressed as mean ± SEM (*n* = 288 for TCPS and *n* = 315 for graphene, **p < 0.01). Li et al. (2011) with permission from Elsevier Ltd., copyright 2011.

Graphene biocompatibility and its effects on the performance of living cells have shown that graphene on top of matrix substrates does not impede the interaction between neurons and substrates, thus maintaining neuronal viability (Fischer et al., 2018). Li et al. (2011) investigated how graphene substrates affect neuritis, an important structure for neural functions, during the maturation of mouse hippocampal cultures compared to tissue culture polystyrene (TCPS) substrates to address the detailed interaction between graphene and tissues/cells in the neural system. The results showed that graphene, as a biocompatible matrix, effectively stimulated neurite sprouting and outgrowth. Neurons started to extend their neurites toward the periphery after being cultured on the graphene substrates (Figure 2A). The phase-contrast micrograph indicates that the original trajectory extended along the neurite to calculate its length. As shown in Figure 2B, neurite numbers in each cell on the graphene substrate significantly increased between 2–7 days, compared with those with TCPS treatment. The neurite lengths also increased with graphene and TCPS, but the average length with graphene was significantly longer than that with TCPS (Figure 2C).

However, *in vivo* studies have demonstrated that the immune system is highly sensitive to graphene and quickly responds by increasing anti-inflammatory cytokine synthesis (Duch et al., 2011; Dudek et al., 2016). Low concentration of graphene, GO and rGO caused serious inflammation in rats (Wang Y. et al., 2011; Schinwald et al., 2012; Chortarea et al., 2022) and perturbed locomotor behavior in an *in vivo* zebrafish model (Cellot et al., 2020). Graphene functionalization is also used to minimize its adverse effects in *in vivo* settings. Fabricating graphene with poly(ε-caprolactone) caused an acceptable degree of immunological response *in vivo* (Wang W. et al., 2019). Hung et al. (2021) fabricated GO with gold and observed incredible immunological compatibility and anti-inflammatory effects *in vivo*. GO coated with biomimetic baicalin effectively ameliorate the inflammatory response *in vivo* (Guo et al., 2021). Furthermore, 500 mg/kg dose of dextran-coated GO had no significant effect on the immune system (Kanakia et al., 2014), and GO-PEG showed normal values of blood hematology analysis (Yang et al., 2013; Chong et al., 2014). Recently, Ding et al. (2020) reported a time- and dose-dependent, stealth-but-activating effect of GO-PEG on macrophages, and a low dosage of GO-PEG resulted in a mild, transient, and bearable immunological response *in vivo*. Feito et al. (2019) demonstrated the mechanism underlying macrophage response to GO-PEG labeled with fluorescein isothiocyanate. GO-PEG labeled with fluorescein isothiocyanate does not cause macrophage polarization and promote the M1/M2 balance, ensuring an appropriate immune response, which is consistent with *in vivo* studies (Lee et al., 2008, 2020; Hung et al., 2021; Zou et al., 2021). Kurapati et al. (2018) showed that graphene could be degraded by myeloperoxidase from active neutrophils *in vivo*. The biodistribution of graphene quantum dots (GODs) in *in vivo* mouse experiments did not accumulate in main organs with fast kidney clearance, probably due to their ultra-small size (Kanakia et al., 2014).

Of note, graphene-based materials in some medical and non-medical fields may exhibit toxicity in human cells, leading to cell death. For example, Jagiełło et al. (2019) investigated the *in vitro* effects of GO and rGO on human umbilical cord Wharton’s jelly-derived mesenchymal stem/stromal cells. GO had no cell toxicity despite its flake size. GO and rGO induced a prominent toxicity phenomenon in a dose- and time-dependent manner. Moreover, GO induced stronger toxicity to the cells than rGO (Wang K. et al., 2011; Kang et al., 2017). Interestingly, rGO with high reduction levels decreased cell proliferation and promoted cell necrosis. A study on graphene toxicity-induced cell necrosis reported that the mechanism underlying graphene toxicity is mainly related to reactive oxygen species production in cells, leading to DNA and protein damage and subsequently cell death through the necrosis pathway (Chatterjee et al., 2015; Sawosz et al., 2015). Functionalization of GO and rGO using different surfactants plays a significant role in reducing toxicity. Wojtoniszak et al. (2012) investigated GO and rGO toxicities using three surfactants, including PEG, sodium deoxycholate, and polyethylene glycol–polypropylene glycol–polyethylene glycol (Pluronic P123). GO functionalized with PEG had the lowest toxicity (36.3%) compared with other surfactants. Cytotoxicity analysis revealed that toxicity depends on the type of surfactants and concentration of nanomaterials to maintain stabilization and avoid agglomeration. Recently, Dybowska-Sarapuk et al. (2020) suggested that reducing the number of functionalized surfactants of graphene to a proper range could reduce the risk of induced cytotoxicity.

rGO injection at centration of 0.004 μg/μL into the core of olfactory bulb *in vivo* does not affect *de novo* neurogenesis and neuronal survival, nor produce immune response (Defteralı et al., 2016a). Intracerebral injection of GO *in vivo* does not induce neurotoxic effects and acute neuroinflammatory in brain structure (Portioli et al., 2020). Intratracheal injection of GO at low concentration in mice caused persistent and severe pulmonary injuries (Duch et al., 2011; Singh et al., 2011). Oral administration of GO at a dose of 0.5–100 μg/mL in *Caenorhabditis elegans* caused irreversible damage to the nervous system, which is closely related to reactive oxygen species production (Wu et al., 2013; Zhao et al., 2018). Overall, toxicity may be attributed to the interaction between some physical and chemical properties of graphene materials, such as the dose, size, shape, type of functional groups, reactive oxygen species production, and administration route (intravenous or oral).

Another popular member of the carbon nanomaterials family, carbon nanotubes demonstrated no major toxicity on cell lines, organotypic slice cultures, and dissociated primary cultures (Lovat et al., 2005; Fabbro et al., 2012; Lee et al., 2015). Similar to graphene, the toxicity of carbon nanotubes is associated with their soluble forms because of improper functionalization. In an *in vitro* study, Simon-Deckers et al. (2008) investigated the toxic effects of multi-walled carbon nanotubes, the most commonly used carbon nanotubes, on A549 human pneumocytes. This study demonstrated that nanotube toxicity, which is stronger than metal oxide nanoparticle toxicity, is mainly triggered by their entrance into cells and dispersion in the cytoplasm, regardless of their length. Similarly, in *in vivo* animal studies of multi-walled carbon nanotubes, exposure was induced through aspiration, inhalation, or intratracheal instillation, thereby inducing interstitial fibrosis and pulmonary inflammation (Porter et al., 2010; Poulsen et al., 2015). Moreover, Rahman et al. (2017) demonstrated that, due to their sizes, multi-walled carbon nanotubes can easily enter the pleural space, interstitium, and highly vascularized alveolar regions, demonstrating high-degree pulmonary biopersistence. These results indicate that the toxicity and safety of graphene must be considered.

Nonetheless, the results from *in vitro* or *in vivo* animal studies cannot be directly applied to humans. Graphene-based materials would be degraded to research tools if they only have good characteristics in experimental settings instead of being surgically applicable. Jakus et al. (2015) established 3D printable graphene fabricated with biodegradable polyester polylactide-co-glycolid containing 20–60% solid content and discovered that it is intraoperatively suitable for surgical procedures in a human cadaver, showing the possibility of graphene bio-ink as a standout candidate among emerging medical devices. However, *in vivo* pre-clinical studies for evaluating biocompatibility and toxicity to establish a detailed standard for elements of graphene-based materials for their potential clinical applications are lacking. In summary, suggestions for overcoming the disadvantages of graphene will promote the future development of graphene-based materials.

## 7. Applications of graphene in the nervous system

### 7.1. Functions of graphene in neurons

Electrical stimulation during the early stage of neuronal development indicates the role of graphene in cell differentiation and phenotypic maintenance (Kam et al., 2009; George et al., 2017). Biomaterial substrates have been investigated for supporting and controlling cellular growth in culture by mimicking natural neural cellular microenvironment. Moreover, various biomolecules can be added to promote neuronal growth and control neuronal survival. Several studies have demonstrated that graphene-based materials can act as biocompatible substrates to enhance neuronal growth, proliferation, and differentiation, as well as neurites sprouting and outgrowth, such as mesenchymal stem cells (MSCs), neural stem cells (NSCs), PC12 cells, dorsal root ganglion (DRG) neurons, retinal ganglion cells, and cortical neurons (Agarwal et al., 2010; Li et al., 2011, 2013; Nayak et al., 2011; Park et al., 2011; Wang et al., 2012, 2019b; Bendali et al., 2013; Sahni et al., 2013; Solanki et al., 2013; Tang et al., 2013; Akhavan and Ghaderi, 2013b; Hong et al., 2014; Shah et al., 2014; Yang et al., 2014; Lee et al., 2015; Bramini et al., 2016; Guo et al., 2016; Rauti et al., 2016; Veliev et al., 2016; Defteralı et al., 2016b; Das et al., 2017; Rastogi et al., 2017; Convertino et al., 2018, 2020b; Kitko et al., 2018; Fang et al., 2019; Fu et al., 2019; Qi et al., 2019; Zhang et al., 2019; Lin et al., 2020; Table 2). These outstanding results are probably the consequence of a complex interplay of electrical, mechanical, and chemical properties imposed by graphene, making it difficult to distinguish the microscopic origin of the effect of graphene on neurons. The topographic cues of graphene with many ripples and wrinkles may resemble the surrounding neural matrix, offering potential substrates for the growth of new neurons. The surface chemistry of graphene plays a part in cellular communication between neurons and substrates. Meanwhile, the electrical conductivity of graphene is widely believed to play a critical role in promoting the outgrowth of neurons and neurites (Cellot et al., 2009; Veliev et al., 2016; Pampaloni et al., 2018). However, a recent study found that both graphene films with high (below 1 kΩ^−1^) and low (up to 70 kΩ^−1^) conductivity favored neuron spreading without clear alteration, indicating that the high electrical conductivity of graphene is not the crucial property and the efficient range of its electric conductivity for graphene-based neuronal interfaces is broader than expected (Capasso et al., 2021). The guidance of neurites induced by the graphene edge may be due to its chemical composition. After *in vivo* implantation or long-term culture for several weeks, the chemical coating would eventually dissolve or degrade, which might expose toxicity concealed by the coating on the surface of the graphene (Bendali et al., 2013).

**Table 2:** The applications of graphene in neural areas.

Applications	Form of graphene	References
Promotes the differentiation of neural stem cells into neurons	Graphene-silicon dioxide nanoparticle mixture scaffold	Yang et al. (2014)
Induces the growth and differentiation of neurons	1. PLLA scaffold coated with GO	Bhang et al. (2013) and Zhang et al. (2010)
	2. Three-dimensional porous graphene scaffolds	Jiang et al. (2016), Zhou et al. (2018), and Javadi et al. (2018)
Boosts nerve cell proliferation	GO nanocomposite multilayer film	Qi et al. (2019) and Zhang et al. (2016)
Electrical stimulation promotes neurite growth	Scaffold materials based on graphene biocompatible conductive PPy and PAn	Ghasemi-Mobarakeh et al. (2011)
Decreases the release of non-specific drugs, improves the targeting efficiency, reduces toxicity	1. Graphene	Chowdhury et al. (2015), Din et al. (2017), and Asghari et al. (2020)
	2. Functionalized GO loaded with pirfenidone	Mendonça et al. (2015) and Yang et al. (2015)
	3. GSPI 409	Wang et al. (2013)
Improve the quality of the nerve repair	Tubular prosthesis combining graphene with PCL 434	Assaf et al. (2017)
Electrical stimulation repairs damaged nerves	PLGA/GO nanocomposite materials 442	Feng et al. (2011) and Fu et al. (2019)
Lead to the pro-inflammatory effects	Two-dimensional graphene	Nezakati et al. (2014) and Song et al. (2022)
Lead to the pro- and anti-inflammatory effects	Three-dimensional graphene	

GO, graphene oxide; PLLA, poly-L-lactide; PPy, polymers polypyrrole; PAn, polyaniline; GSPI, silica-coated graphene nanosheet; PCL, poly (ε-caprolactone); PLGA, poly (lactic-co-glycolic acid).

### 7.2. Chemical vapor deposition-grown graphene

Zhang et al. (2010) found that graphene sheets synthesized via the radio frequency catalytic CVD approach induced cytotoxic effects on neuronal PC12 cells in a concentration and shape-dependent manner. Time-dependent caspase3 activation after exposure to CVD-grown graphene at 10 μg/mL dosage shows evidence of apoptosis. However, Nayak et al. (2011) found CVD-grown graphene did not hamper hMSCs proliferation and accelerated their differentiation. Graphene film enhances the differentiation of hNSCs toward neurons (Park et al., 2011). CVD-grown graphene under low pressure promotes the adhesion and proliferation of hippocampal neurons but does not induce cell stress (Rastogi et al., 2017).

### 7.3. Graphene oxide and its derivative

GO can stimulate embryonic stem cells and NSCs to differentiate into neurons as well as promote the differentiation and growth of neuronal axons (Kim T. H. et al., 2013; Guo et al., 2017). GO exhibits a size-dependent effect on the differentiation of mouse NSCs (Lin et al., 2020) and hippocampal cells (Rauti et al., 2016). Some studies reported that GO impaired the excitatory transmission of primary and hippocampal neurons (Bramini et al., 2016; Rauti et al., 2016, 2019). However, Secomandi et al. (2020) found that GO decreased the cultured amygdalar neurons and transiently increased their excitatory transmission. Further experiments should be conducted to demonstrate the controversy over these different neurons after the GO application. Conductive materials for neural cells have been exploited, such as polyaniline, polythiophene, poly-3,4-ethylene dioxythiophene, and polypyrrole (Lei et al., 2014). Several studies functionalized GO with these conductive materials to enhance conductivity (Deng et al., 2011; Zhang and Zhao, 2012; Shah et al., 2014; Fu et al., 2019). Conductive graphene-based composites have been successfully applied to improve recording with substantial SNR and accelerate electrical stimulation of neural cells. Nearly all experiments were conducted on the graphene-based substrates for 1–3 weeks. This procedure is useful for deciphering the long-term effects of these substrates on neurons. However, neurites sprouting and hippocampal neuron outgrowth cultured on the substrates increased maximally during the second day of observation, which is consistent with the results of Convertino et al. (2020b), reaching a non-significant level compared with the control culture. Future studies should focus on exploring the early development of neurons on the substrates and determining the elements that influence neurites outgrowth based on culture time. In addition, the degradation effect of graphene-based substrates on neurons should be investigated.

Interestingly, few studies have investigated how neurons respond to graphene and its derivates. The nanoscale mechanisms underlying graphene-induced neurite outgrowth of neural cells remain unknown. Graphene can increase the local accumulation of nerve growth factors to boost the elongation of peripheral neurons (Convertino et al., 2020a). In previous studies, graphene increased the level of growth-associated protein-43, an efficient marker of axon outgrowth, in both hippocampal neurons (Li et al., 2011) and PC12 cells (Wang et al., 2019b), suggesting that central and peripheral neurons cultured on graphene-based substrates may be driven by the same mechanisms of neuritogenesis. Conductive materials enhance the cellular bioelectric property, through which electrical stimulation can facilitate and control neuritis outgrowth and axonal elongation (Uesaka et al., 2005; Meng, 2014). Moreover, the hippocampal neurons demonstrated increased cell firing, and this change might be due to the alteration of the membrane ion currents by the material interface (Pampaloni et al., 2018). Differentiated NSCs showed upregulated genes involved in the calcium signaling pathway, such as G protein-coupled receptors and Na/Ca exchangers (Park et al., 2011), and increased spontaneous Ca^2+^ spiking (Tang et al., 2013). Thus, primary neurons exposed to GO flakes demonstrated impaired Ca^2+^ signaling and several proteins associated with intracellular trafficking (Rauti et al., 2016). Moreover, cellular neurogenesis may be induced by graphene via focal adhesion kinase and p38 mitogen-activated protein kinase cascades (Lee et al., 2015), along with the activation of ERK1/2 phosphorylation (Lin et al., 2020). In addition, downregulating phosphatidylserines, which regulates the negative charge of the cytosolic side of the membranes, and upregulating phosphatidylethanolamines, which comprise membrane phospholipids of a synaptic vesicle, affect their fission and fusion (Gaffaney et al., 2008; Morita et al., 2012). The alteration in phosphatidylethanolamines/phosphatidylserines ratio may be involved in the change in synaptic transmission described previously. These studies are in the early stage; despite the different techniques of synthesizing graphene and various biological cells studied, the interaction between graphene and neural cells should be explored further for a better understanding and use of its biological efficacy. Overall, the understanding of graphene is advancing, providing a solid foundation for neuroscience research and the clinical application of graphene-based materials, given the advancement of biological tissue engineering and material science.

### 7.4. Drug delivery and graphene treatment for neurological diseases

Drug delivery to the brain may be ineffective for treating central tumors because the central nervous system is complex, increasing disease severity and major side effects without achieving satisfactory therapeutic effects (Meng et al., 2017; Fernandes et al., 2018; Saeedi et al., 2019). The current treatment modalities, including chemo- and photothermal-theory, have low efficacy and various side effects and risks and lack specific targeting. Nanocarriers are now superior approaches to cancer therapy. Graphene has been exploited as a drug loading material with effective potency and efficiency because of its π–π stacking, high specific surface, and hydrophobic and electrostatic interactions. Graphene and its derivatives have been developed into vectors of genes and drugs with functionalization as drug delivery systems because of its advantageous properties in achieving targeting and local delivery.

**Figure 3:**
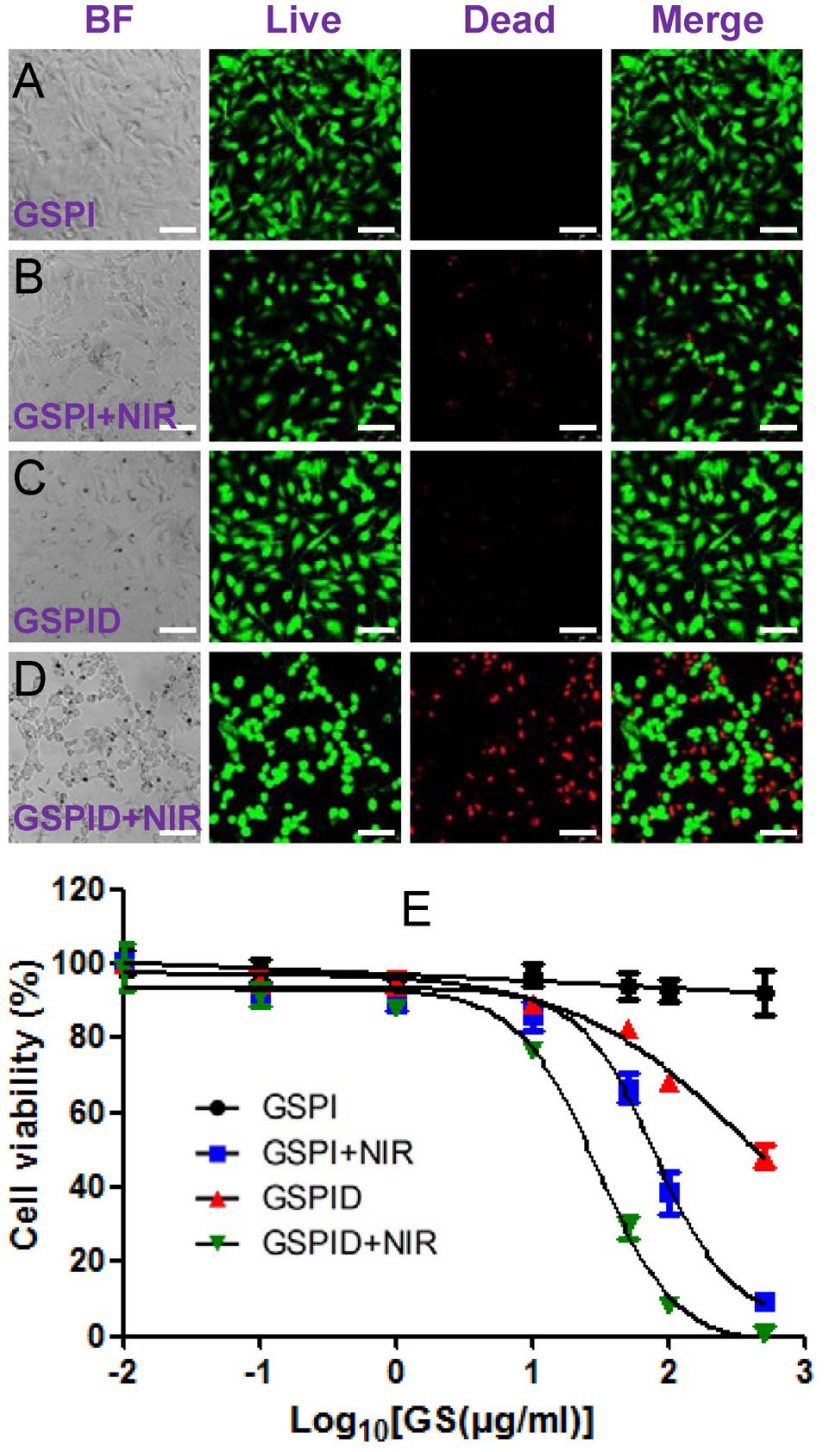
**(A–E)** Qualitative results of confocal microscopy showing the viability of glioma cells by LIVE-DEAD staining. BF, bright field images, Green: live cells. Red: dead cells; Wang et al. (2013) with permission from ACS Publications, copyright 2013.

Graphene is a mode for intracellular administration. GO and rGO can deliver antigens that modify innate immune cells to trigger effective and adaptive immune responses for immunotherapy *in vivo* (Sinha et al., 2019; Wang X. et al., 2019; Parker et al., 2022). Yang et al. (2022) revealed that GO with antigen can be efficiently taken up by dendritic cells via receptor-mediated endocytosis, which enhances immune responses and increases delivery effectiveness both *in vitro* and *in vivo*. Meanwhile, Yan et al. (2019) loaded rGO with IDO inhibitor, which can trigger immune responses, and successfully induced antitumor immunity. Fang et al. (2022) assembled GO and metal–organic framework, Fe-porphyrin, of which the porous structure and large surface area enabled drug delivery. The use of a metal–organic framework as a photosensitizer under laser irradiation enables effective synergistic treatment through PTT. Moreover, a study functionalized graphene with PEI to deliver drugs. PEI is a non-viral gene carrier that can interact with negatively-charged DNA or RNA to form a complex. Synthesis of PEI-GO nanocomplex via π-π stowage and hydrophobic interaction produced higher transfection efficiency and less toxicity than PEI alone, delivering gene and achieving a high-drug load of insoluble drugs (Huang et al., 2016). These drug-delivery systems can improve targeting efficiency through functionalization with targeted genes (Chowdhury et al., 2015; Keles et al., 2016; Din et al., 2017; Asghari et al., 2020). For example, glioblastoma multiforme (GBM), a neurogenic tumor, is difficult to treat because of the intracranial location of the tumor. Jaworski et al. (2013, 2015) discovered that apoptosis occurred in GBM cells cultured on graphene and rGO platelets, indicating the possible effectiveness of graphene in anticancer therapy. Arginine-producing nitric oxide can affect reactive oxygen in mitochondria, cause cell apoptosis (Jeong et al., 2010), and have a high affinity for graphene (Hughes and Walsh, 2015). Sawosz et al. (2015) added arginine to rGO and examined the anticancer effect of the complex on GBM cells. They revealed that adding arginine to rGO increased rGO activity and led to GBM cell apoptosis. Another study used GO and rGO to transport microRNA to GBM cells. These complexes had less harmful effects on the viral carrier, but more efficiency of microRNA delivery than the viral carrier. GO and rGO functionalized with microRNA-induced microRNA deregulation stress, regulated relative gene expression to apoptosis, and increased GBM cell apoptosis (Kutwin et al., 2021). Wang Y. et al. (2013) designed an initial drug-delivery system that used chemo-photothermal treatment to target GBM cells. Coating graphene nanoparticles with mesoporous silica improved super absorption under NIR and drug loading efficiency compared with graphene nanosheets alone. A targeting peptide was designed and exploited as a GBM-cell targeting ligand; it modified mesoporous silica nanoparticles. Loading doxorubicin onto the targeting peptide conjugated-mesoporous silica covered-graphene system caused the specific and highest death rate of GBM cells with near-infrared irradiation than single photothermal- or chemotherapy (Figures 3A–E).

**Figure 4:**
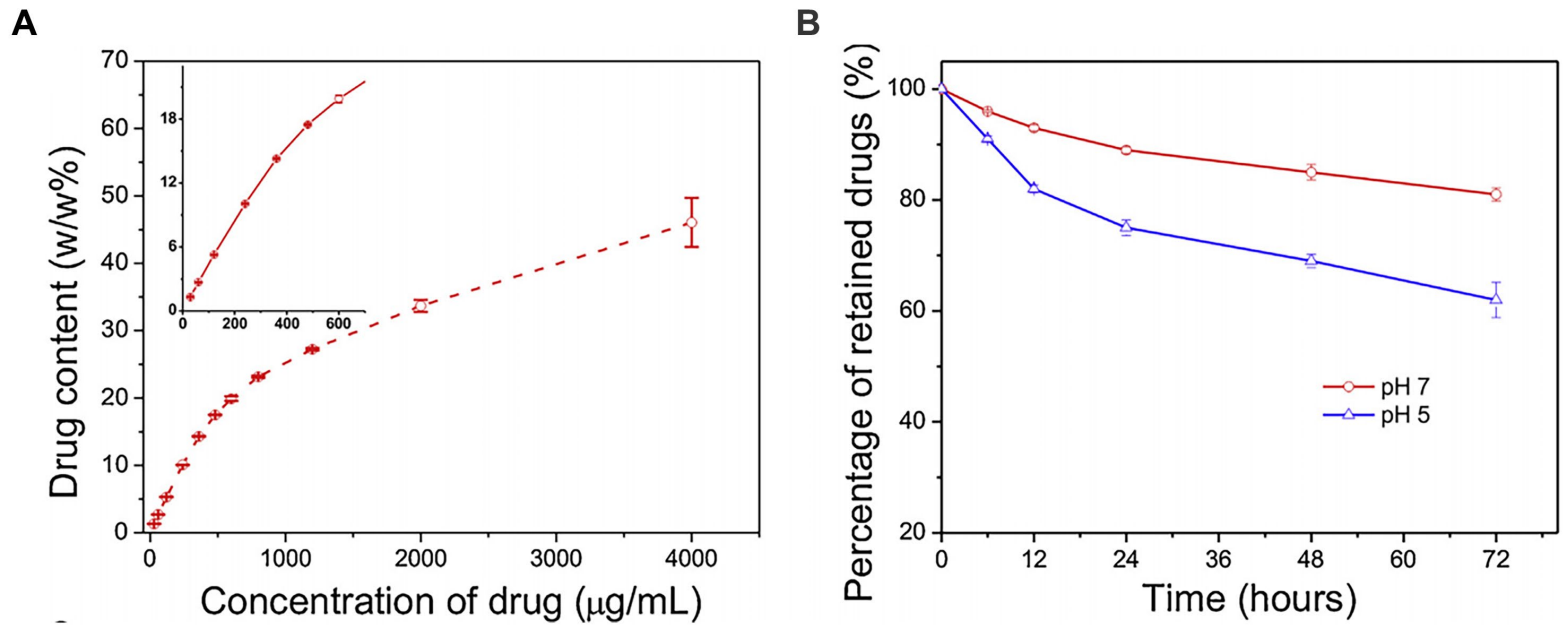
**(A)** The content of FGO loaded with pirfenidone was determined at the concentration of the drugs. **(B)** Graph of the release of pirfenidone from FGO by pH 5 and pH 7 in in a specific time point; Yang et al. (2015) with permission from Elsevier Ltd., copyright 2015.

Two points must be considered when nanocarriers deliver drugs to the central nervous system. First, drugs must have the ability to penetrate the blood–brain barrier (BBB) to ensure the delivery of sufficient dosage to kill cancer cells. Second, the delivery should specifically target cancer cells, avoiding harmful effects on normal tissues. For example, subarachnoid hemorrhage occurs in severe neurological diseases, and studies have demonstrated the feasibility of using functionalized GO (FGO) for effective drug delivery in such cases. The content of FGO nanotablets loaded with pirfenidone, which is used to treat subarachnoid hemorrhage, gradually increases with an increase in drug concentration (Figure 4A), making FGO a suitable medicine carrier that can be loaded with pirfenidone. The functionalization of transcription activator peptide and methoxy PEG introduced onto GO nanosheets aid BBB crossing and improve drug stability in blood circulation. Transcription activator peptide improved nanosheet ability to cross the BBB. Moreover, long-chain polyethers of methoxy PEG prolonged the lifetime of pirfenidone in circulation and increased the permeability of nanomaterials through blood vasculatures. Furthermore, pirfenidone was released from FGO at a pH of 5 within 72 h and not at a pH of 7 (Figure 4B). Subarachnoid hemorrhage may trigger an inflammatory response, and the inflammatory microenvironment is an acidic site with a pH of 5.5. Therefore, the effective release of pirfenidone amount under acidic conditions indicates that the FGO drug-delivery system has a satisfactory therapeutic effect on inflammatory lesions (Yang et al., 2015). The functionalization of graphene-based nanomaterials improves BBB penetration ability, increasing drug concentration gradients inside and outside blood vessels, and facilitating drug entry into the brain through capillary endothelium, providing a good carrier for targeted drug delivery in the brain. Similarly, Dong et al. (2016) examined the efficacy of targeting drug delivery with chemo-photothermal therapy on rats with GBM. Loaded onto transferring-conjugated GO-PEG, doxorubicin entered through the BBB and accumulated in the GBM region of the brain. Under NIR radiation, the tumor temperature significantly increased as the drug-delivery system is absorbed because of the GO’s aromatic structure. This photothermal effect could be mediated by applying the drug delivery later. The life of rats bearing GBM was also prolonged with targeted drug delivery utilizing nanomaterials. These characteristics indicate an admirable drug-delivery system for treating diseases. The saturable light absorption of graphene may also be useful for targeted delivery of improved graphene and its derivatives, which are accumulated in cancer cells after intravenous administration in treating neurogenic tumors. Thus, irradiating the tumor with NIR light will lead to the photothermal ablation of tumor cells, while retaining ordinary tissue with ambient temperature (He et al., 2016).

Furthermore, neurodegeneration diseases of the nervous system, including AD and PD, lack accurate and economic clinical diagnostic tools. No drugs have successfully treated these diseases in clinical settings. Therefore, associated biomarkers must be detected and quantified for diagnosing these diseases early. One of the most important contributions to neurological diseases is the aggregation of amyloid fibrils. Graphene-based materials have been extensively explored to not only detect amyloid protein sensitively and quantitatively but also prevent the accumulation of amyloid, which might provide new insights into alternative diagnostics and therapy at the early stage of AD and PD. The conformation of Aβ peptides produced by β-secretase changes from alpha-helix to beta-sheet to create insoluble fibrils, within which heavy metal ions eventually result in amyloid plaques (Kayed et al., 2003). Beta-amyloid_1-42_ (Aβ_1-42_) is an important biomarker in AD and PD (He et al., 2018). Lopes et al. (2014) first demonstrated the progress of aggregating Aβ_1-42_ absorbed on the graphite electrode via its tyrosine oxidation record using differential pulse voltammetry. This experimental method offered a potential tool for detecting and preventing Aβ aggregation. Yousaf et al. (2019) reported a probe based on bovine-serum-albumin-capped fluorine functionalized GQDs that enabled spontaneous fluorescence-based monitoring of Aβ_1-42_ *in vitro* with a 10 times higher detection rate than that of conventional thioflavin dye and effective detection of amyloid plaques *in vivo*. Nangare and Patil (2022) decorated GO with chitosan, which offers sites for Aβ_1-42_ antibodies via layer-by-layer assembly on the surface of the plasmon resonance biosensor. This inimitable feature of biosensor achieved detection with a linear range of 2 fg/mL–400 ng/mL and a limit of 1.21 fg/mL. Yu et al. (2021) used a silver probe and magnetic GO substrate capturing the antigen, which was formed into a typical sandwich structure, thus achieving a detection linear range of Aβ_1-42_ from 100 pg. mL^−1^ to 10 fg mLd^−1^. Such a tool was successfully used and verified on AD human serum samples. However, the reported antibody against oligomers would possibly target monomers. Graphene-based electrochemical detection with a high specificity could also depend on DNA hybridization apart from antigen–antibody interactions. Bonanni et al. (2012) first examined the ability of GO to conjugate with DNA hybrids and discovered its promising role in DNA labeling. Song et al. (2022) conjugated GO with Aβ_1-42_-specific aptamer to generate a novel biosensor that could detect Aβ_1-42_ of 0.1 to 10 uM through surface-assisted laser desorption ionization mass spectrometry. MiRNA-137 was identified as a reliable biomarker for AD (Azimzadeh et al., 2017). Chang et al. (2021) modified GO with gold nanostar through an amine linker to capture miRNA-137 on the complementary sequence, achieving a detection limit at 10 fM with 1 fM sensitivity. The graphene surface can highly transfer the fluorescence quenching efficiency, making it a suitable broad-spectrum fluorescence quencher (Ma et al., 2017). Rahaie and Noroozi (2019) applied GO with SYBR green based on the hybridization chain reaction for detecting miR-137 and demonstrated that this designed biosensor achieved sensitive detection ranging from 0.05 nM to 5 nM with a limit at 82 pM. Moreover, Zhou et al. (2018) offered a strategy based on GO and entropy-driven strand displacement reaction. In this experiment, researchers released the bound Aβ_1-42_ oligomers using hairpin structure DNA probes, which hybridized with the fluorescein-labeled complementary probes. Continuous cycle reactions amplified the fluorescence signal until GO started to absorb the excess labeled probes. This method ensured ultrasensitive detection of Aβ_1-42_ oligomers at limit of 20 pM.

Inhibiting fibrillation is a prominent strategy for preventing or treating neurodegenerative diseases (Haataja et al., 2008). Graphene and its derivatives modulate the pattern of fibril formation. Gregory et al. (2020) showed that graphene effectively mitigates amyloid fibrillation using hen egg-white lysozyme, a classical model of amyloid-forming protein, using the interfacial charge transfer associated with hydrophobic and π-π interactions. This result was consistent with the discovery made by Ban et al. (2018) using graphene oxide quantum dots. Baweja et al. (2015) also discovered that GO and rGO decreased the beta-strand propensity of Aβ amino acid residues, inhibiting alpha helix to beta-sheet transition of Aβ peptide and the electrostatic interactions contributed to its adsorption on GO and rGO. However, researchers have suggested that the oxidative degree of GO surface influences the interplay between materials and filament growth. He et al. (2019) demonstrated that non-uniform GO in oxidation sites exhibited a remarkably stronger perturbation on Aβ fibril. Another study revealed that high oxidative GO-20 and GO-40 inhibited fibril elongation, while low oxidative GO-10 accelerated fibril elongation. The details of how the oxidation degree of GO causes different effects in *in vitro* experiments will provide new insights into the design strategy for amyloidosis therapy. A density functional theory study recently demonstrated that GO with 12.5% oxygen can effectively reduce Aβ plaque in AD (Liu and Luo, 2021). In *in vivo* experiments, Xiao et al. (2016) conjugated GODs with peptide glycine-proline-glutamate and administrated it to APP/PS1 transgenic mice. The deposition area of Aβ plaque reduced in the GODs with peptide glycine-proline-glutamate group compared with the control group. Besides, the learning and memory capacity was enhanced. Tak et al. (2020) synthesized GODs from the *Clitoria ternatea* flower and discovered that GODs effectively reduced AD-like symptoms in rodents. Many results support that graphene-based materials can be used as promising agents for treating neurodegenerative disease; however, there are limitations to consider. For example, several studies have demonstrated an apparent breakdown of Aβ fibril, and the resultant protein was not exclusively a monomer, raising concerns about the potential toxicity of the remaining smaller soluble oligomers. Further studies should explain the fate of these smaller oligomers in cells. The effect of graphene-based material concentration on neurodegenerative symptoms *in vivo* should also be determined.

In this review, we emphasized the potential, specific, and bright prospects of graphene for treating nervous system diseases. In the absence of ideal treatments for neurological diseases, such as brain cancer, AD, and PD, graphene-based nanotechnology can be used as a drug carrier to better control drug release, thereby improving drug delivery to the specific site and a tool to diagnose and treat these diseases. This technology should be further developed as matured gene or drug-targeted delivery systems and combined with physicochemical methods to obtain more satisfactory treatment results and provide a solid foundation for future neurotherapy development (Liao et al., 2018; Yao et al., 2019).

### 7.5. Graphene in nerve repair

The ability of graphene to repair nerves has been evaluated in several studies. The large surface area of graphene can increase its adsorption performance and act as an interface material for nerve repair (He et al., 2016; Domínguez-Bajo et al., 2017). For example, peripheral nerve injuries cause disconnections between spinal neurons and targeted organs. Autografting is a widely used approach if end-to-end neurorrhaphy is infeasible. With the recent developments in nanotechnology, Jakus et al. (2015) demonstrated that graphene-based nerve scaffold has great *in vivo* biocompatibility, as previously described. However, the nerve conduits were only implanted subcutaneously for 30 days, with no further neural expression performances. Moreover, Assaf et al. (2017) fabricated a tubular prosthesis made of graphene and polycaprolactone and examined the efficiency of the material on sciatic nerve repair in rats with disconnection. The prosthesis improved the number of myelinated axons in repaired nerves 12 weeks after surgery and contributed to a satisfactory regeneration outcome after a peripheral nerve injury. Furthermore, a relatively low mass ratio of the anterior tibialis muscle was observed, indicating the possibility of muscle atrophy. In addition, Qian et al. (2018b) conducted more detailed examinations to evaluate the long-term effects of graphene-related nerve conduit in sciatic nerve restoration, including counting myelinated axons, average myelinated axon diameter, the thickness of myelin sheath, and regenerated axon area. With the tubulization of the sciatic nerve defect in rats at 18 weeks after surgery, using polydopamine- and arginylglycylaspartic acid modified- and polycaprolactone functionalized-graphene via 3D printing fabrication, and layer-by-layer casting method, four parameters in the tubulization group demonstrated no distinct difference compared with those in the autograft group. In addition, the locomotor and sensory function recovery were examined. The conduit group loaded with cells also showed similar results as the autograft group, indicating that muti-layered 3D graphene can reverse muscle atrophy. Thus, these results demonstrated that graphene-based nanoscaffolds can cure long-term peripheral nerve defects by promoting axonal regeneration and nerve function recovery. Graphene foam/hydrogel scaffolds significantly promote the recovery of sciatic nerves *in vivo* with no obvious organ lesions or damage (Huang et al., 2022). Qian et al. (2018a) demonstrated that the GO/PCL nerve guidance conduit also successfully repaired sciatic nerve defects in rats, effectively promoting its functional and morphological recovery, and showed no obvious toxicity for the long-term. Loading graphene with brain-derived neurotrophic protein that contributes to axon pathfinding and neuronal migration can enhance the ability of graphene-based material in nerve repair. Huang et al. (2021) loaded a graphene/hydrogel scaffold with netrin-1, an axonal guidance cue, and successfully supported sciatic nerve regeneration. The performance of these kinds of graphene is superior to that of an autologous graft. Moreover, Pan et al. (2019) further immobilized PLGA/GO and brain-derived neurotrophic factor and insulin-like growth factor-1, which are involved in nerve regeneration, and demonstrated that PLGA/GO fabricated with brain-derived neurotrophic factor and insulin-like growth factor-1 significantly boosted the functional locomotor recovery and the counting of neurons located at the injury sites in animal models of spinal cord injury (SCI). GO-modified poly(D,L-lactide-co-caprolactone) significantly accelerates the functional recovery of sciatic nerve 8 weeks post-operation *in vivo* (Zhang et al., 2020). These results suggest that engineered graphene-based materials provide a novel therapeutic approach to cure SCI as a nerve implant.

Moreover, macrophage recruitment increases after nerve injury, highlighting the importance of immune responses in tissue regeneration. In the context of neural tissues, several studies have investigated the effect of graphene-based scaffolds for nerve repair on immune cells, which are important for driving wound healing and tissue regeneration. López-Dolado et al. (2016) demonstrated that implanting 3D rGO scaffolds in SCI rats induced immune modulation and angiogenic responses without causing systemic toxicity. *In vivo* studies using SCI rats showed that implanting 3D rGO scaffolds in the injured spinal cord improved the structured lesion zones more than those left untreated, indicating the contribution of scaffolds in improving sealing and injury stabilization. Pro-regenerative macrophages were more obviously visible at the interface and functional new blood vessels inside the scaffolds than lesion regions without scaffolds 30 days after surgery (López-Dolado et al., 2015, 2016). The immune signature may guide NSCs migration. Furthermore, whether graphene could elicit immune responses and how it affects NSC behaviors when interfaced with macrophages remain unclear. Thus, Jiang et al. (2016) demonstrated that 3D graphene evoked mild neuro-immunity and caused macrophages to secrete some inflammatory cytokines without changing their cell cycle profiles and viability. These results were consistent with the findings of Serrano et al. (2018), which showed that 3D graphene culture interfaced with macrophages promoted neurosphere formation and NSC migration from the neurospheres. In contrast, 2D graphene and TCPS interfacing with macrophages failed to achieve such results. Hence, the capacity of 3D graphene to govern NSCs migration might depend on its topographical structure by mildly activating macrophages.

The fabrication of graphene-based composite nanofibers can determine their function, providing versatile therapeutic approaches. Wang et al. (2019a) fabricated PLGA/GO nanofibers using methylene blue via physisorption, which is used to activate the autophagy signaling pathway and regulate the function of neural progenitor cells. They discovered that neural progenitor cells cultured on the PLGA/GO loaded with methylene blue entered a quiescence phase to avoid apoptosis and diminish tau phosphorylation. Unlike similar GO fabrication into various patterns through microcontact printing, Min et al. (2017) synthesized GO and magnetic nanoparticles, which were used as a force, to transfer the GO film into the desired substrate. A mixed mode of GO driven by magnetic force can precisely guide the formation of synaptic connections between neurons in different directions, providing a different method for curing SCI.

Graphene plays an indispensable role in various neuroscience applications by promoting nerve regeneration and neural restoration and treating some nerve diseases. Factors that promote the repair of neurite injury sites may vary with cell type. Therefore, more studies are required to overcome these problems and transform the successful repair of damaged nerves into functional recovery in clinical treatment (Liu and Jan, 2020).

## 8. Conclusion

Carbon nanomaterials have gained attention because of their large surface area, mechanical stability, electrical conductivity, and biocompatibility. Graphene family materials have been studied and used in several technical and scientific fields, including neurobiomedicine. In this review, we sketched the role of nanostructured carbon materials (particularly graphene) in neuroscience in *in vitro* and *in vivo* studies. A summary of the properties of graphene, including its mechanical and electrical properties, optical and thermal properties, biocompatibility, and toxicity, revealed that the characteristics of graphene and its derivatives vary from size, structural dimensions, and layer number-to-surface chemistry. Summarizing the applications of graphene and its derivatives, including neural culture, drug delivery, diagnosis and treatment of neuronal diseases, and nerve repair (Table 3), we found that their applications depend on the differences in functionalization, purification, and chemical and morphological formations. Therefore, many discrepancies exist between experimental results owing to various involved elements, such as potential toxicity and induced immune responses, which directly determine the possibility of further bioapplication. Fortunately, such experimental outcomes have increased enormously with great progress in scientific and clinical fields.

**Table 3:** The function of graphene in neurons.

Substrate	Coating	Culture system	Days in culture	Effects	Reference
Graphene	None	Mice dorsal root ganglion neurons	3	Graphene significantly promoted axonal elongation and dampen neuronal spiking.	Convertino et al. (2020a)
GO	None	Mouse neural stem cells	24 h	GO triggered positive neuronal differentiation via phosphorylation of ERK1/2 with the downregulation of TRPC2 gene.	Lin et al. (2020)
Graphene	None	neural stem cells	5	Cells proliferation increased along with metabolic reconfiguration.	Fang et al. (2019)
poly(lactic-co-glycolic acid)/GO	None	Mice neural stem cells	7	Neuronal differentiation, proliferation significantly increased with significant neurite elongation.	Fu et al. (2019)
poly(lactic-co-glycolic acid)/GO	insulin-like growth factor 1	Neural stem cells	7	NSCs survival, proliferation, and differentiation remarkably enhance.	Qi et al. (2019)
Graphene	Silk fibroin	PC12 cells	4	Cell spreading and differentiation were promoted. And neurites outgrowth significantly increased by 74.5%.	Zhang et al. (2019)
Graphene	Silk fibroin and hydrogels	PC12 cells	6	Neurite-related gene expression markers, including GAP43 (growth associated protein 43) and SYP (synaptophysin) increased. Therefore, the composite hydrogels with graphene improved the PC12 cell proliferation, differentiation and neurites growth.	Wang et al. (2019)
Graphene	None	Retinal ganglion cells	7	Graphene reduced the number, but increased basal activity, of functional cation channels of retinal ganglion cells with great neurites outgrowth.	Fischer et al. (2018)
Graphene	None	Rat dorsal root ganglion neurons	17	There were dense axonal networks on coated graphene with neurites outgrowth	Convertino et al. (2018)
Graphene	None	Rat hippocampal neurons	8–10	Graphene modulated the distribution of extracellular ions at the interface with neurons to affect neuronal excitability.	Pampaloni et al. (2018)
Graphene	None	Rat hippocampal neurons	12–18	Graphene increased cell membrane cholesterol and potentiate neurotransmission. And the increase of neurites was small.	Kitko et al. (2018)
Ink Graphene	None	mesenchymal stem cells	3	Cellular differentiation and paracrine activity significantly enhanced.	Das et al., 2017
Graphene	None	Rat hippocampal neurons	9	Cell adhesion and proliferation were promoted. And graphene did not affect the mitochondrial morphology, mitochondrial membrane potential or the autophagy levels.	Rastogi et al. (2017)
Graphene	None	Mouse hippocampal neurons	5	Neuronal attachment, outgrowth and axonal specification increased.	Veliev et al. (2016)
GO	Poly-L-Lysine	Hippocampal neurons	7	GO down-regulated neuronal synaptic signaling without affecting cell viability.	Rauti et al. (2016)
Graphene	Laminin	Rat neural stem cells	7	The alteration of passive and active bioelectric properties of NSCs was accompanied by the increased their differentiation by graphene. Furthermore, graphene promoted synapse proteins expression, spine density and synaptic activity.	Guo et al. (2016)
Thermally reduced graphene	None	Mouse neural stem cells	4–15	The morphological differentiation was favored. Moreover, it promoted the long survival of neurons.	Defteralı et al. (2016b)
Graphene	Poly-D-lysine	Rat cortical neurons	14	The viability, morphology and functionality of neurons unchanged.	Bramini et al. (2016)
GO	Poly-D-lysine	Rat cortical neurons	14	The viability of cortical neurons did not change. However, excitatory transmission was impaired with enhanced inhibitory transmission and it was companied by alteration of Ca^2+^ dynamics.	Bramini et al. (2016)
Graphene	Polymethyl methacrylate	Human neuroblastoma cells	7	Neurite outgrowth enhance with upregulation of a key genes of cell neurogenesis, neurofilament light chain.	Lee et al. (2015)
GO	None	Mouse embryonic stem cells	10	Dopamine neuron differentiation enhanced and related gene expression also increased.	Yang et al. (2014)
polycaprolactone/GO	None	Neural stem cells	7	Selective guidance of NSCs differentiation toward oligodendrocyte increased.	Shah et al. (2014)
Graphene	polymethyl methacrylate	Rat PC12 cells	7	Neural cells proliferation and neurite outgrowth enhanced.	Hong et al. (2014)
Graphene	Laminin	Human neural stem cells	14	NSCs differentiation increased with enhanced spontaneous firing activity. But neurite number unchanged.	Tang et al. (2013)
GO/ silica nanoparticles	None	Human neural stem cells	14	Neuronal differentiation enhanced and axons growth increased.	Solanki et al. (2013)
Graphene	None	Adult retinal ganglion cells	6	Adult neurons can survive and grown neurites.	Bendali et al. (2013)
Graphene	Laminin	Mouse neural stem cells	5	NSCs proliferation increased with upregulation of Ki67 expression.	Li et al. (2013)
Graphene/TiO_2_	None	human neural stem cells	21	Neurons differentiated significantly increased.	Akhavan and Ghaderi (2013a)
Graphene	None	Rat cortical neurons	20	Pristine graphene supported neurons survival, growth and adhension. Robust growth of neurites was observed.	Sahni et al. (2013)
Graphene	Poly-D-lysine and laminin	Retinal ganglion cells	3–6	Neuronal viability and cell adhension enhanced. Neurites sprouting increased.	Bendali et al. (2013)
Graphene	Fluorine	Mesenchymal stem cells	7	Mesenchymal stem cells proliferation and differentiation increased	Wang et al., 2012
Graphene	Laminin	Human neural stem cells	3–21	NSCs differentiation enhanced and neurites outgrowth also increased.	Park et al. (2011)
Graphene	Poly-L-lysine	Mouse hippocampal neurons	2–7	Neurite sprouting and outgrowth significantly enhanced and expression of growth- associate protein-43 also increased.	Li et al. (2011)
Graphene	Polymethyl methacrylate	Human mesenchymal stem cells	15	Human mesenchymal stem cells differentiation remarkably increased	Nayak et al. (2011)
rGo	Poly-L-lysine	PC12 cells	5	PC12 cells proliferated well and neurites extended and outgrew.	Agarwal et al. (2010)

GO, graphene oxide; rGO; reduced graphene oxide; PC12 cells, pheochromocytoma 12 cells; NSCs, neural stem cells; TRPC, transient receptor potential canonical; TiO_2_, titanium dioxide.

The observed discrepancies in *in vitro* and *in vivo* studies suggest that current studies have several limitations. First, compared with the number of *in vitro* studies, *in vivo* studies exploring the basic properties of graphene, specifically its biocompatibility and toxicity are limited. Lack of consistency between *in vitro* and *in vivo* results may downgrade graphene into only a research tool. Regarding *in vivo* study models, nearly all animal models are limited to mice, and no other species have been considered in exploring graphene applications. Second, most studies suggest that nanomaterials induce a mild immune response. However, researchers are yet to identify the standard method of administration. Furthermore, whether nanomaterials act the same way when eliciting an immune response via different routes of administration *in vivo* remains unknown, although the toxicity and pharmacokinetics of nanoparticles are closely dependent on the administration route. Third, most studies, whether *in vitro* or *in vivo*, did not perform in-depth investigations on the underlying mechanism involved in the relationship between cell differentiation and graphene-based substrates, such as induced morphologic interplay and cellular modulation pathways. Without these fundamental definitions, determining the role of graphene in biomedical applications may be challenging.

The following are proposals for future experiments for improving graphene and its derivatives development: First, the main consideration of nanomaterials in biomedical application is their biocompatibility. Further studies must focus more on biocompatibility and toxicity *in vivo* because the results from *in vitro* studies cannot be directly applied to humans. Moreover, the graphene-based implant as an electrode or scaffold should also be further investigated to observe its long-term effect *in vivo*. More animal models should be considered to clarify the relationship between different species and graphene. Second, graphene can exhibit long-term and short-term toxic effects on the same cell and can potentially cause long-term damage to cellular structures. This review highlighted that graphene toxicity is highly dependent on excretion, functionalization, size, and structural dimensions. Therefore, comparing graphene toxicity between different experiments is difficult, given its diversity in shape, surface, size, and fabrication. Thus, the fabrication and terminology of graphene and the examination of toxicological methodologies must be standardized. This will better aid the understanding of the physicochemical properties and potential toxicity of graphene *in vivo* and *in intro*, allowing its practical application in humans. Third, future studies should incorporate graphene with various optical components to probe deeper brain tissues and understand how brain circuits process information because a graphene electrode with a two-photon microscope can record the whole cortical surface in neuroscience investigation. Moreover, electrical stimulation has been clinically applied in rehabilitation after nerve injury. Graphene-based conduits have shown great potential in nerve repair as scaffolds. Future studies should measure the potential of combining electrical stimulation and graphene-based conductive scaffolds in clinical nerve repair.

This review sheds light on the safety and biological properties of graphene that must be considered before the utilization of graphene-based therapy for human benefit. This may pave the way for using graphene and its derivatives as powerful materials with versatile scientific and clinical applications.

## Author contributions

TY and YY wrote the first draft of the manuscript. L-YM and YL wrote sections of the manuscript. JB, F-YW, and LZ reviewed the literature. All authors contributed to manuscript revisions as well as read and approved the submitted version.

## Funding

This work was supported by the National Natural Science Foundations of China (81560231; 81860251; 51703192), the Innovation Team Project of Yanbian University (China), and the International Cooperation Project of Science and Technology Department of Jilin Province (20210402004GH).

## Conflict of interest

The authors declare that the research was conducted in the absence of any commercial or financial relationships that could be construed as a potential conflict of interest.

## Publisher’s note

All claims expressed in this article are solely those of the authors and do not necessarily represent those of their affiliated organizations, or those of the publisher, the editors and the reviewers. Any product that may be evaluated in this article, or claim that may be made by its manufacturer, is not guaranteed or endorsed by the publisher.

## Abbreviations

2D: two-dimensional
DNA: deoxyribonucleic acid
RNA: ribonucleic acid
PTT: photothermal therapy
AD: Alzheimer’s disease
PD: Parkinson’s disease
SNR: signal-to-signal ratio
3D: three-dimensional
IR: infrared
NIR: near-IR
GO: graphene oxide
*rGO: reduced graphene oxide
PEG: polyethylene glycol
PEI: polyethyleneimine
Aβ: amyloid β-protein
TCPS: tissue culture polystyrene
DRG: dorsal root ganglion
GAP-43: growth-associate protein-43
GBM: glioblastoma multiforme
BBB: blood–brain barrier
FGO: functionalized GO
SCI: spinal cord injury
NSCs: neural stem cells
